# Medical Students’ Views About Having Different Types of Problem-Based Learning Tutors

**DOI:** 10.1007/s40670-018-00634-9

**Published:** 2018-11-14

**Authors:** Shobhana Nagraj, Susan Miles, Pauline Bryant, Richard Holland

**Affiliations:** 1 The George Institute for Global Health, Nuffield Department of Women’s & Reproductive Health, University of Oxford, Le Gros Clark Building, South Parks Road, Oxford, OX1 3QX UK; 2 Norwich Medical School, University of East Anglia, Norwich, NR4 7TJ UK; 3 Centre for Medicine, Leicester Medical School, University of Leicester, Leicester, LE1 7RH UK

**Keywords:** Problem-based learning, Peer-assisted learning, Near-peer tutoring, Undergraduate medical education

## Abstract

**Background:**

At Norwich Medical School, Year 3 or 4 medical students taking a year out of the 5-year undergraduate MBBS degree to do a master’s degree in clinical education worked as near-peer problem-based learning (PBL) tutors for students in Year 2. Peer-assisted learning has been shown to benefit both peer tutors and tutees; in this study, experiences of students with near-peer PBL tutors were compared to students with other types of PBL tutor.

**Methods:**

Using existing student evaluation data, we compared student views about PBL tutor performance, PBL group functioning, and overall satisfaction with PBL learning experience according to whether their PBL tutor/s were (1) a single near-peer tutor (later-year MB BS student), (2) a single staff tutor, (3) multiple staff tutors, or (4) multiple newly qualified doctor tutors.

**Results:**

Results indicated that students’ evaluation of tutor performance was more positive for near-peer PBL tutors compared to both groups of staff tutors for most areas evaluated. Additionally, students’ evaluation of overall satisfaction with PBL was more positive for near-peer PBL tutors compared to multiple staff tutors. Tutor performance for multiple staff tutors was evaluated less positively compared to both single staff and multiple newly qualified doctor groups. But there were no statistically significant differences between the four groups regarding PBL group functioning.

**Conclusion:**

Near-peer PBL tutors perform comparably or better to staff PBL tutors in salient measures of tutor performance and group functioning. We conclude that medical students find near-peer PBL tutors to be an acceptable addition to the PBL tutor workforce.

## Introduction

The General Medical Council (GMC) in the UK have emphasised the importance of medical students taking opportunities to develop their teaching skills during undergraduate medical training [1]. Peer-assisted learning is one way of aiding students in the development of important teaching skills, preparing students for their future role as educators after they become qualified doctors [2].

Peer-assisted learning has been used in a variety of different ways in medical education, including problem-based learning (PBL). Whilst PBL is a student-led learning method, facilitation of the PBL process is aided by the presence of an effective PBL tutor, whose main roles are to guide students in the activation of their prior knowledge, signpost students to discover and build upon this knowledge, and facilitate effective group interaction and functioning.

Peer-assisted learning in PBL has been utilised both through peer tutoring (where a student from the PBL group takes on the tutor role) [3, 4] and near-peer tutoring (where a senior student acts as tutor) [5–8]. In some studies, the near-peer tutor has co-tutored with a staff tutor [5, 6].

The benefits to both tutors and tutees of peer-assisted learning in PBL present a mixed picture. Studies have found no differences between peer- and faculty-tutored groups in achievement in exams, or self-assessment of skills or own performance in the group [3–5]. Similarly, studies have found no meaningful differences between peer- and faculty-tutored groups regarding perception of tutor performance and group functioning [3, 6]. Such findings could lead to the conclusion that peer tutoring in PBL is at least as effective as faculty tutoring. However, Steele et al. [3] also found that whilst the results of focus groups indicated students favoured peer-tutored groups, viewing such groups to be ‘more co-operative, more efficient and less stressful’ (p. 26) than faculty-tutored group, students felt that faculty input would lead to less anxiety about accuracy of knowledge for complex cases. Kassab et al. [4] found that students struggled more with initially defining and understanding the problem when in peer-tutored groups. Problematically, observation of peer-tutored groups found students taking shortcuts to speed up the PBL process, leading to concerns that students might not be learning reasoning skills for approaching clinical problems [3]. More encouragingly, peer-tutored PBL groups are judged to have a more relaxed atmosphere, to lead to a more meaningful learning experience and more useful group work [4, 5]. Furthermore, students rate peer PBL tutors more positively than faculty tutors with regard to providing feedback and having a better understanding of student difficulties [4]. These mixed findings indicate that there remains a need to investigate peer-assisted learning in PBL specifically.

At our institution, Norwich Medical School (NMS), we have been using PBL as a key learning modality throughout all 5 years of the undergraduate medical curriculum since the MB BS course started in 2002. Across 12 taught modules, there are over 100 PBL groups requiring tutors. Recruiting sufficient tutors for all 5 years of the programme has often raised staffing issues. Peer teaching has been identified as one way to reduce the teaching demand pressure on faculty [2]. We first piloted, and subsequently expanded a programme involving near-peer PBL tutors across Year 2 of the medical curriculum to ascertain if near-peer PBL tutors would be an effective alternative to standard staff PBL tutors. We present the findings of student evaluations of the programme here.

## Methods

### Introduction of Near-Peer PBL Tutors

Near-peer tutors were first used in PBL at NMS in 2012–2013, originally drawing on self-selected students who were studying for an intercalated medical degree. Such students had completed their third or fourth year of medical study at NMS then intercalated to undertake a locally taught postgraduate (master’s degree) qualification in clinical education before continuing with the MB BS. As part of these studies, students were required to gain some direct experience within the education setting, e.g. working as a demonstrator in our anatomy laboratory, a facilitator for the interprofessional skills training, or a PBL tutor. Initially, numbers of intercalating students were small; however, in recent years, numbers of students intercalating and expressing interest in becoming a near-peer PBL tutor have increased and the programme has expanded to accommodate nine and 13 PBL tutors over the last two academic years, respectively (2015–2016 and 2016–2017). Simultaneously, a formal application and selection process was introduced to select the most appropriate students to be near-peer tutors. Selection is based on academic performance, good professional standing, and a statement of interest in being a near-peer PBL tutor. All PBL tutors, including near-peer PBL tutors, attend a compulsory one-day training programme which covers the following: adult learning theory, examples of good teaching and learning, theoretical underpinnings of PBL, the role of the PBL tutor in the session, the role of the PBL tutor outside the session, key challenges in PBL sessions, how to undertake peer review, and giving and receiving feedback. In addition, near-peer PBL tutors also attend a session around maintaining confidentiality and professional boundaries. An experienced staff PBL tutor acts as a mentor to the near-peer PBL tutors. The mentor is available to provide support and guidance with any issues the near-peer PBL tutors encounter during their year of tutoring. All near-peer tutors will have experienced PBL as a medical student for a minimum of 3 years. Near-peer PBL tutors will take the same group of 10 students for the whole academic year. Year 2 was selected as the most appropriate year for the near-peer PBL tutors to be introduced as it ensured that the near-peer tutors would not be tutoring students in their own year group or the year group they would be returning into when they continued their MB BS studies. Near-peer PBL tutors are remunerated for their time acting as a PBL tutor.

### Evaluating Student Experience of PBL

Each academic year, all medical students at NMS are required to complete an ‘Annual Evaluation’ through which student feedback is collected on all aspects of the MB BS course. Students are sent an email link to the evaluation form via their university email address with up to three email reminders to non-responders during the data collection period. Within the evaluation, students are asked to rate on a 5-point Likert scale four questions about their PBL group experience, six questions on their views about aspects of the facilitation provided by their PBL tutor, and one question rating their overall satisfaction with their PBL learning experience (Appendix, Box 1). Higher scores indicate a more positive evaluation. Additionally, students have the opportunity to provide free text comments about their PBL tutor, the most useful aspects of the PBL process that year, and what could be improved about the PBL process for the future. The closed questions focus on areas previously identified as salient to the students’ PBL experience through analysis of open-ended comments received about effective tutoring and learning environments in earlier Annual Evaluations. The areas identified by students were also judged to be important for a successful PBL learning experience by the faculty Lead for PBL at the time, the MB BS Course Director, and experienced PBL tutors. As such, it was determined that questions about these areas would form a suitable question set for evaluating the students’ PBL experience, with the purpose of improving tutor performance and student learning experience in the group. Thus, following each Annual Evaluation, PBL tutors are provided with an individual report of the anonymous feedback from the students in their PBL group (mean scores for the closed questions, and raw comments for the open-ended question about PBL tutor performance), with average scores across all PBL groups who have just studied the same module for comparison purposes. PBL tutors use this feedback to develop their tutoring skills where needed across the areas of tutor performance and group functioning judged to be salient by students and senior faculty for a successful PBL learning experience under the NMS approach to PBL.

All students complete the Annual Evaluations each academic year so as to contribute to the ongoing development of the MB BS. Students are asked to provide consent for their evaluation data to also be used for further purposes, including journal publications of evaluation findings. Data from four academic years, 2012–2016, were included in this analysis.

## Results

### PBL Group Characteristics

There were 17 Year 2 PBL groups in the 2013–2014 and 2014–2015 academic years and 18 PBL groups in 2012–2013 and 2015–2016, with a total of 670 students across the 4 years. Of these students, 598 (89%) consented for their data to be used for research purposes. One PBL group in 2012–2013 had both a staff and a near-peer PBL tutor and was therefore excluded from all analyses (8 consenting students), leaving a final number of 590 students (88% response rate) in 69 PBL groups (Table 1). The number of students providing feedback on each PBL tutor ranged from 5 to 10 with a median of 9.

**Table 1:** PBL groups and tutor types by academic year

Academic year	Number of groups					Number of students	
	Total number of PBL groups	With a near-peer tutor	With a single staff tutor	With multiple staff tutors	With newly qualified doctor tutors	Total number of students	Number of students who consented
2012–2013	18*	1 (+ 1 shared group)*	12 (+ 1 shared group)*	2	2	168	145
2013–2014	17	2	11	2	2	166	144
2014–2015	17	3	7	3	4	167	152
2015–2016	18	9	7	1	1	169	157
Total	70	15	37	8	9	670	598

*One group (*n* = 8 students) had a single staff PBL tutor and a near-peer PBL tutor; this group is included here in this table, but was excluded from all analysis reported in this paper, making the total number of consenting students included in the paper 590

There were 235 (39.83%) male students and 355 (60.17%) female students. The students’ ages ranged from 17 to 42 years, with a mean of 19.27 years (standard deviation of 2.21).

Of the 590 students, 128 had a near-peer PBL tutor (a later-year MB BS student currently intercalating to take a master’s degree) and 462 had a non-peer PBL tutor. Of the latter, 314 students had one staff member tutoring them for the whole academic year, 70 had more than one staff member tutoring them during the academic year (e.g. a different tutor for each of the three modules in the year, usually for reasons of staff availability), and 78 had more than one newly qualified doctor tutoring them during the academic year (these were fully qualified doctors who had completed their undergraduate medical degree and were now in their second year of postgraduate medical training on the Foundation Programme working in academic posts; like the multiple staff tutors, each Foundation doctor took the students for one of the three modules in the year). This gave four PBL tutor-type groups for comparison: ‘near-peer PBL tutor’, ‘single staff PBL tutor’, ‘multiple staff PBL tutors’, and ‘multiple newly qualified doctor PBL tutors’.

### Statistical Analysis

Students’ experiences with aspects of PBL, evaluated by the 11 individual questions outlined in Box 1, were compared by the four PBL tutor types. The non-parametric Kruskal-Wallis test for comparing multiple unrelated groups was used for the analysis as the data were interval, the samples sizes for the four PBL tutor-type groups differed, and the variances were dissimilar. The Kruskal-Wallis test was conducted on the data for each of the 11 questions separately, with a corrected significance level of 0.0045 for multiple tests (Bonferroni correction, 0.05/11 = 0.0045). In combination with inspection of the mean ranks for the Kruskal-Wallis test, the non-parametric Mann-Whitney *U* test was performed for post hoc analysis of the questions where the Kruskal-Wallis test indicated that there was a significant difference between the four PBL tutor-type groups to identify where the differences lay. All analyses were conducted in SPSS version 22.

### Differences Between the Four PBL Tutor-Type Groups

The Kruskal-Wallis tests indicated that there were no significant differences between the four PBL tutor-type groups for any of the four PBL group performance questions.

In contrast, there were significant differences between the four PBL tutor-type groups for all six of the PBL tutor performance questions and for the overall satisfaction with PBL question (Appendix Box 2), as such post hoc testing using the Mann Whitney *U* test was conducted (using a corrected significance level of 0.05/42 = 0.0012; each one of the four PBL tutor-type groups was compared with the other three types, on seven individual questions) to ascertain which groups differed from each other.

**Fig. 1:**
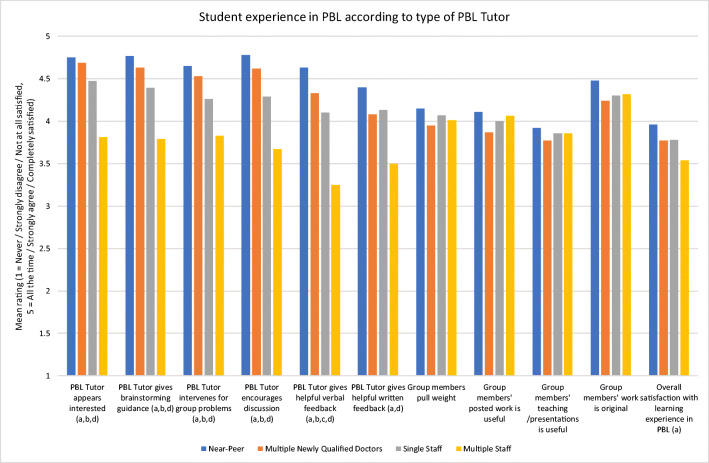
Mean ratings for all of the student evaluation of PBL questions by PBL tutor type. **a** Near-peer PBL tutor groups significantly higher than multiple staff PBL tutor groups. **b** Near-peer PBL tutor groups significantly higher than single staff PBL tutor groups. **c** Near-peer PBL tutor groups significantly higher than multiple newly qualified doctor PBL tutor groups. **d** Single staff and multiple newly qualified doctor PBL tutor groups both significantly higher than multiple staff PBL tutor groups

It was found that students in the near-peer PBL tutor groups gave higher scores for all six of the PBL tutor performance questions and the overall satisfaction with PBL question when compared to the multiple staff PBL tutor groups, and for five of the PBL tutor performance questions when compared to the single staff PBL tutor groups. But they gave higher scores for just one PBL tutor performance question (verbal feedback from the PBL tutor) when compared to the multiple newly qualified doctor PBL tutor groups (Fig. 1).

In addition to the lower scores for all PBL tutor performance questions and the overall satisfaction with PBL question when compared to the near-peer PBL tutor groups, students in the multiple staff PBL tutor groups also gave lower scores for all six of the PBL tutor performance questions when compared to the single staff and multiple newly qualified doctor PBL tutor groups.

## Discussion

Our results indicate that student experience with near-peer PBL tutors was better for most domains of student experience with their PBL tutor compared to both single and multiple staff PBL tutors.

The theoretical basis for the success of peer-assisted learning may rest on two main premises: the concepts of cognitive congruence and social congruence [9]. Cognitive congruence relates to the peer teachers’ sharing or being able to relate to the knowledge base and requirements of the students, thus enabling them to express themselves using the students’ language, thereby making it easier for students to grasp difficult concepts. Social congruence refers to peer teachers’ ability create a positive learning environment where students feel safe to show ignorance and make mistakes, through communicating empathetically and informally with students and showing interest in them [2, 9, 10].

It has been argued that through their recent experience with the course, near-peer PBL tutors have a sensitivity to the needs of students [5]. They display more socially and cognitively congruent behaviour such as better understanding of student difficulties and their practical needs regarding assessments, creating a more relaxed atmosphere within the group and being more interested in the students’ experiences [4, 11]. As Year 3 and 4 students on the MB BS, the near-peer tutors in this study are acutely aware of the PBL process from the student learning perspective and what is required of students in Year 2, having recently experienced it themselves. This ‘insider’ knowledge may give them more legitimacy in the PBL facilitation process in the eyes of the students. Near-peer PBL tutors being perceived as appearing interested, providing appropriate guidance, intervening when the group was experiencing problems, encouraging sufficient group discussion, and providing helpful verbal feedback to a higher degree than both groups of staff PBL tutors, and providing helpful written feedback to a higher degree than multiple staff PBL tutors may be explained by the more congruent behaviour on the part of the near-peer PBL tutors in these areas.

Another possible explanation for these findings is that peer tutors may put greater effort into, and spend more time on, preparing for the PBL tutorial. It takes considerable time to review each week’s PBL case, accompanying learning material and the documents prepared by students about the learning objectives they have identified, and also to provide feedback to students about their performance; staff may not have the time they would ideally like to dedicate to this around their other teaching, research and clinical commitments. Additionally, the topics are directly relevant to the near-peer tutor’s own learning needs and so they may view time spent with the PBL material as a valuable period of revision for their next year of study and future assessments. We do not, however, have any data relating to preparation times of PBL tutors to substantiate this explanation. It is also not clear from this study whether peer tutors are displaying other behaviours which may lead to increased student satisfaction, such as helping students with exam preparation, advice, and study tips although responses to the open-ended question regarding PBL tutor performance suggest that this is happening in addition to facilitation of the PBL session.

Ratings for the newly qualified doctor tutors sit between the higher rated near-peer tutors and the single staff tutors. Newly qualified doctors as tutors represent an example of ‘cross-level’ peer teaching, that of recently graduated doctors teaching medical students [9]. Researchers have found that the quality of teaching sessions and perceived learning from such sessions is the same for near-peer and cross-level teachers [12, 13]. However, characteristics such as enjoyment of the sessions, relevance of teaching to the students’ needs, and delivery of teaching have been found to favour near-peers in contrast to peers at a further distance; this has been attributed to reduced congruence between junior doctors and medical students [12, 13]. As such, it is possible that our findings are attributable to the newly qualified doctor tutors having less congruence than the near-peer tutors, but more congruence than the staff tutors.

Near-peer, newly qualified doctor and single staff PBL tutors were rated more highly than multiple-staff PBL tutors. The reasons for this are unclear. Whilst it could be argued that this finding reflects students’ valuing a continuity of the tutor relationship over the course of the academic year, all of the newly qualified doctor tutor groups also had multiple tutors in the year. This makes explanations based on the amount of time tutors spend with the student during the year, or the number of tutors facilitating each group less plausible. It is possible that staff tutors who share a group are less committed to the PBL process for some reason. Alternatively, it may be that a closer social and cognitive congruence exhibited by the newly qualified doctor tutors (compared to the staff tutors) counters some issues related to having multiple tutors.

There were no statistically significant differences between the four groups relating to the PBL group performance questions, suggesting that these characteristics of the PBL experience are somewhat independent of the tutor characteristics evaluated in this study. It is notable that for all tutor types, the group experience questions resulted in lower scores than the tutor questions. Thus, all tutor types need to help their students develop in these areas.

### Limitations and Future Research

Whilst student perceptions of their learning environment are important, the evaluation data used in this study are subjective and may not accurately reflect objective measures of tutor performance or adherence to PBL principles during their sessions. All PBL tutors at NMS are required to undergo a PBL training day to encourage uniformity of PBL facilitation methods across tutors, and tutors are additionally required to have one peer observation of their PBL tutoring every academic year. There are, however, currently no objective measurements made of tutor performance during PBL at NMS. In the future, it would be worthwhile undertaking research to identify the specific behaviours of highly rated PBL tutors in each of the four groups of tutors through observation, and seeing how these behaviours match to expected facilitation methods and other effective tutor attributes.

There is inevitably some variation between tutors within each of our four groups. Therefore, although near-peer PBL tutors as a group are rated highest by students, variation amongst each tutor-type group means that not all individual near-peer tutors compare more favourably to all individual staff tutors. An observational study of near-peer and faculty tutoring in PBL groups revealed significant variations in tutor practices within groups of near-peer and faculty tutors; this within-group variation was greater than the variation across the tutor types [12]. Thus, it would be important to take account of individual differences in facilitation skills if planning to introduce near-peer PBL tutors and ensure that training meets the needs of all prospective tutors.

Finally, student satisfaction with their PBL tutor may not equate with improved learning. We have not linked these findings to performance in any of our assessments, or actual clinical performance (as exams are inevitably a surrogate for that performance). Generally, students in peer-tutored PBL groups have achieved similar outcomes in assessments [3–5], but the picture is not clear [14] and more research is needed in this area to ensure that peer tutoring in PBL results in at least equivalent outcomes to the faculty tutoring it would be replacing.

Additional important future research would include investigating near-peer PBL tutors’ experiences of PBL as a tutor. Such research could elucidate the benefits for tutor’s continuing undergraduate training when they return to their course and generalizable skills for future practice as a doctor. It could also identify any areas where they struggled, for example shifting their role from student to tutor on their own course of study or developing facilitating skills without slipping into teaching. Such information would help to develop training for future near-peer tutors.

## Conclusion

Our study has shown that medical students are as satisfied with the performance of group members and more satisfied with PBL tutor performance when facilitated by near-peer PBL tutors as compared to staff tutors. Additionally, satisfaction with overall PBL learning experience was higher for students with near-peer tutors compared to multiple staff tutors. As such, from a student experience perspective, our findings indicate that near-peer PBL tutors, when carefully selected, trained, and supported, can be an acceptable addition to faculty PBL tutors, thus helping to ease pressure on teaching staff and freeing them up to perform other educational activities.

## Appendix

Box 1 Student evaluation of PBL, questions asked in the Annual Evaluation

**Table:**

**PBL Tutor Questions:** **(Responses:*****1 = Never, 2 = Rarely, 3 = Sometimes, 4 = Most of the time, 5 = All the time)*** 1. The PBL Tutor appears interested in the group’s learning 2. The PBL Tutor provides appropriate guidance during brainstorming as to the work the group needs to cover each week 3. The PBL Tutor intervenes appropriately when the group is experiencing problems with group dynamics and / or individual students 4. The PBL Tutor encourages sufficient group discussion around the PBL scenario to help my understanding of the week’s topic **(Responses:*****1 = Strongly disagree, 1 = Disagree, 3 = Neither agree nor disagree, 4 = Agree, 5 = Strongly Agree)*** 5. I received helpful verbal feedback from the PBL Tutor 6. I received helpful written feedback from the PBL Tutor on my PBL Tutor Report **PBL Group Questions:** **(Responses:*****1 = Never, 2 = Rarely, 3 = Sometimes, 4 = Most of the time, 5 = All the time)*** 7. All members of the group pull their weight 8. PBL work posted on Blackboard by group members is useful for my learning needs 9. The teaching / presentations of topics provided by group members during the PBL session are useful for my learning needs 10. Work prepared by members of the group for PBL is original, and not just copied and pasted from other sources **Overall Satisfaction with PBL:** **(Responses:*****1 = Not at all satisfied, 2 = Slightly satisfied, 3 = Moderately satisfied, 4 = Very satisfied, 5 = Completely satisfied)*** 11. Please rate how satisfied you have been with your learning experiences in PBL so far this year	

Box 2 Results of the Kruskal-Wallis tests, with mean (standard deviation) ratings for each evaluation question by PBL tutor type

**Table:**

Questions	Near-peer PBL tutor	Single staff PBL tutor	Multiple staff PBL tutors	Multiple newly qualified doctor PBL tutors	Chi-square (df), significance
**PBL Tutor Questions:**					
PBL tutor appears interested^a,b,d^	4.75 (0.55)	4.47 (0.72)	3.81 (0.82)	4.69 (0.52)	80.964 (3), 0.0001
PBL tutor provides appropriate guidance for brainstorming^a,b,d^	4.77 (0.53)	4.39 (0.82)	3.79 (1.03)	4.63 (0.61)	66.981 (3), 0.0001
PBL tutor intervenes when the group is experiencing problems^a,b,d^	4.65 (0.64)	4.26 (0.90)	3.83 (1.02)	4.53 (0.66)	44.526 (3), 0.0001
PBL tutor encourages sufficient group discussion^a,b,d^	4.78 (0.50)	4.29 (0.88)	3.67 (1.13)	4.62 (0.61)	77.025 (3), 0.0001
Helpful verbal feedback from the PBL tutor^a,b,c,d^	4.63 (0.52)	4.10 (0.98)	3.25 (1.32)	4.33 (0.57)	72.088 (3), 0.0001
Helpful written feedback from the PBL tutor^a,d^	4.40 (0.76)	4.13 (0.89)	3.50 (1.06)	4.08 (0.88)	39.535 (3), 0.0001
**PBL Group Questions:**					
Group members pull their weight	4.15 (0.68)	4.07 (0.76)	4.01 (0.73)	3.95 (0.70)	4.981 (3), ns
PBL work posted by group members is useful	4.11 (0.73)	4.00 (0.69)	4.06 (0.80)	3.87 (0.80)	5.821 (3), ns
Teaching/presentations by group members is useful	3.92 (0.76)	3.86 (0.86)	3.86 (0.91)	3.77 (0.76)	1.795 (3), ns
Work prepared by group members is original	4.48 (0.59)	4.30 (0.73)	4.32 (0.70)	4.24 (0.72)	6.847 (3), ns
**Overall Satisfaction with PBL:**					
Overall satisfaction with PBL^a^	3.96 (0.78)	3.78 (0.76)	3.54 (0.77)	3.77 (0.82)	15.571 (3), 0.001

^a^Near-peer PBL tutor groups significantly higher than multiple staff PBL tutor groups

^b^Near-peer PBL tutor groups significantly higher than single staff PBL tutor groups

^c^Near-peer PBL tutor groups significantly higher than multiple newly qualified doctor PBL tutor groups

^d^Single staff and multiple newly qualified doctor PBL tutor groups significantly higher than multiple staff PBL tutor groups

*ns*, not statistically significant
